# Role of high-density EEG (hdEEG) in pre-surgical epilepsy evaluation in children: case report and review of the literature

**DOI:** 10.1007/s00381-021-05069-z

**Published:** 2021-02-18

**Authors:** Michela Quintiliani, Federico Bianchi, Filomena Fuggetta, Daniela Pia Rosaria Chieffo, Antonia Ramaglia, Domenica Immacolata Battaglia, Gianpiero Tamburrini

**Affiliations:** 1 Infantile Neuropsychiatry, Fondazione Policlinico Gemelli IRCCS, Rome, Italy; 2 Pediatric Neurosurgery, Fondazione Policlinico Universitario A. Gemelli IRCCS, Largo F. Vito 1, 00168 Rome, Italy; 3 Clinical Psychology Unit, Fondazione Policlinico Gemelli IRCCS, Rome, Italy; 4 Institute of Radiology, Fondazione Policlinico Gemelli IRCCS, Rome, Italy; 5 Università Cattolica del Sacro Cuore, Rome, Italy

**Keywords:** HD-EEG, Epilepsy, Pediatric, ESI

## Abstract

**Introduction:**

Electrical source imaging (ESI) and especially hdEEG represent a noninvasive, low cost and accurate method of localizing epileptic zone (EZ). Such capability can greatly increase seizure freedom rate in surgically treated drug resistant epilepsy cases. Furthermore, ESI might be important in intracranial record planning.

**Case report:**

We report the case of a 15 years old boy suffering from drug resistant epilepsy with a previous history of DNET removal. The patient suffered from heterogeneous seizure semiology characterized by anesthesia and loss of tone in the left arm, twisting of the jaw to the left and dysarthria accompanied by daze; lightheadedness sometimes associated with headache and dizziness and at a relatively short time distance negative myoclonus involving the left hand. Clinical evidence poorly match scalp and video EEG monitoring thus requiring hdEEG recording followed by SEEG to define surgical target. Surgery was also guided by ECoG and obtained seizure freedom.

**Discussion:**

ESI offers an excellent estimate of EZ, being hdEEG and intracranial recordings especially important in defining it. We analyzed our results together with the data from the literature showing how in children hdEEG might be even more crucial than in adults due to the heterogeneity in seizures phenomenology. The complexity of each case and the technical difficulties in dealing with children, stress even more the importance of a noninvasive tool for diagnosis. In fact, hdEEG not only guided in the presented case SEEG planning but may also in the future offer the possibility to replace it.

## Introduction

Epilepsy surgery represents a valid treatment in drug-resistant epilepsy [2] showing higher rate of seizure freedom and better behavioral outcomes and quality of life, compared to non-surgical cohorts [6, 7]. Multimodal pre-surgical workup is important to identify epileptogenic zone (EZ) [18], to assess complication rates and seizure freedom probability [3]. Phase I investigations include seizure semiology, high resolution MRI [4], video scalp EEG, and neuropsychological assessment [19]. When these results are concordant and EZ is not in eloquent cortex, surgery can be offered. Otherwise, additional investigations, like temporal lobe volumetry and hippocampal relaxometry, are recommended before phase II intracranial recordings. Functional MRI (fMRI) or WADA test are useful in determining language hemisphere dominance [11, 21]. Electrical source imaging (ESI) by high-density EEG (hdEEG) was proved to be a non-invasive, low-cost, and accurate method of localizing the source of interictal and ictal EEG signals [14]. Moreover, ESI provides non-redundant information with respect to phase I assessments, leading to a change in clinical management in one third of patients [9]. Furthermore, ESI with hdEEG has research implications in localizing eloquent cortex as well as in the understanding of brain networks [13, 14, 23].

Analyzing the pertinent literature, the authors found how the increasing interest in hdEEG as ESI enhancer seems to be limited to adult population. Interestingly, few papers address specifically pediatric population even though it is there where it could have the highest value. In fact, children may suffer of more complex forms of epilepsy and in them its prompt resolution can greatly modify short and long-term prognosis.

## Case description

The patient is a 15-year-old boy whose clinical history started at 5 years old. Seizures at onset were (1) imbalance and cold sensation and (2) limbs and face paresthesia followed by loss of consciousness and limbs stiffening. MRI scan showed a right parietal dysembryoplastic neuroepithelial tumor (DNET). After surgical removal, the boy was seizure free for 2 years. At 7 years old, focal seizures characterized by behavioral arrest, mental confusion, left upper limb paresthesia, and distorted voice sensations, followed by lower limb stiffening and weakness or imbalance appeared. Long-term video EEG monitoring showed background rhythm asymmetry and right centro-parieto-temporal interictal paroxysmal abnormalities. Ictal EEG highlighted large slow right parietal and temporal waves preceded by rapid activity. Seizures control lacked despite poly-antiepileptic drugs (AEDs) with valproate acid, oxcarbazepine, and clobazam. Control MRI excluded tumor regrowth showing only scar tissue on the superficial and deep-middle inferior parietal region extending to the marginal and angular gyrus, to the posterior insula, and to the inferior parietal gyrus behind rolandic cortex. Thus, the child underwent a second surgery, aided by electrocorticography (ECoG) to remove the altered sulci in the postero-medial portion of the previous surgical cavity as well as the right superior temporal gyrus. Seizure freedom was achieved for 3 years.

Nonetheless, seizure relapsed after 2 years becoming heterogeneous and multidrug-resistant. The boy experienced anesthesia and loss of tone in the left arm, twisting of the jaw to the left and dysarthria accompanied by daze, lightheadedness sometimes associated with headache and dizziness, and negative myoclonus involving the left hand. Therefore, a new pre-surgical epileptic work up was proposed.

### Long-term monitoring video EEG

The registration was made through 21 copper disc electrodes according to the International System 10-20 (band pass 1.600–70 Hz, sampling rate 512 Hz). The recording lasted 3 days and included Intermittent Light Stimulation protocol at increasing frequencies (3–50 Hz) and 5 minutes hyperventilation. Data were analyzed using Micromed System View. Interictal EEG was characterized by slow activity and spike and slow wave discharges on right central parietal regions, spreading on the posterior vertex ones. Independent and isolated spikes on right posterior temporal derivations were also observed. Three types of seizures were recorded: focal motor, characterized by discharges of spike-wave on the right parietal regions with subsequent bihemispheric diffusion; focal non-motor, characterized by delta-like slow activity; and spike-wave on the right centro-parietal regions with right hemispheric diffusion and negative myoclonus, characterized by slow wave discharges on the right parietal and frontal regions with diffusion on the contra lateral homologous regions.

### Neuropsychological evaluation

Cognitive assessment was carried out using the Wechsler Intelligence Scale for Children (version IV, 2003) which highlighted a normal level (IQ 88), characterized by a disharmonic profile, in the presence of a significant difference (> 12 points) between the indices, to the detriment of the score obtained in working memory (73) and processing speed (82). Visuo-spatial memory was quite impaired (recall of Rey figure—5ds). Verbal memory tests showed normal performance in short-term recall and slight difficulty in the long-term one. Furthermore, there was a marked difficulty in lexical retrieval with a phonological facilitator and slight difficulty with a semantic facilitator. Tests performed with Developmental Neuropsychological Assessment (NEPSY-II, 2007) showed a marked deficit in sustained attention and a slight difficulty in visual-motor integration.

### Electrical source imaging of IEDs

**Fig. 1:**
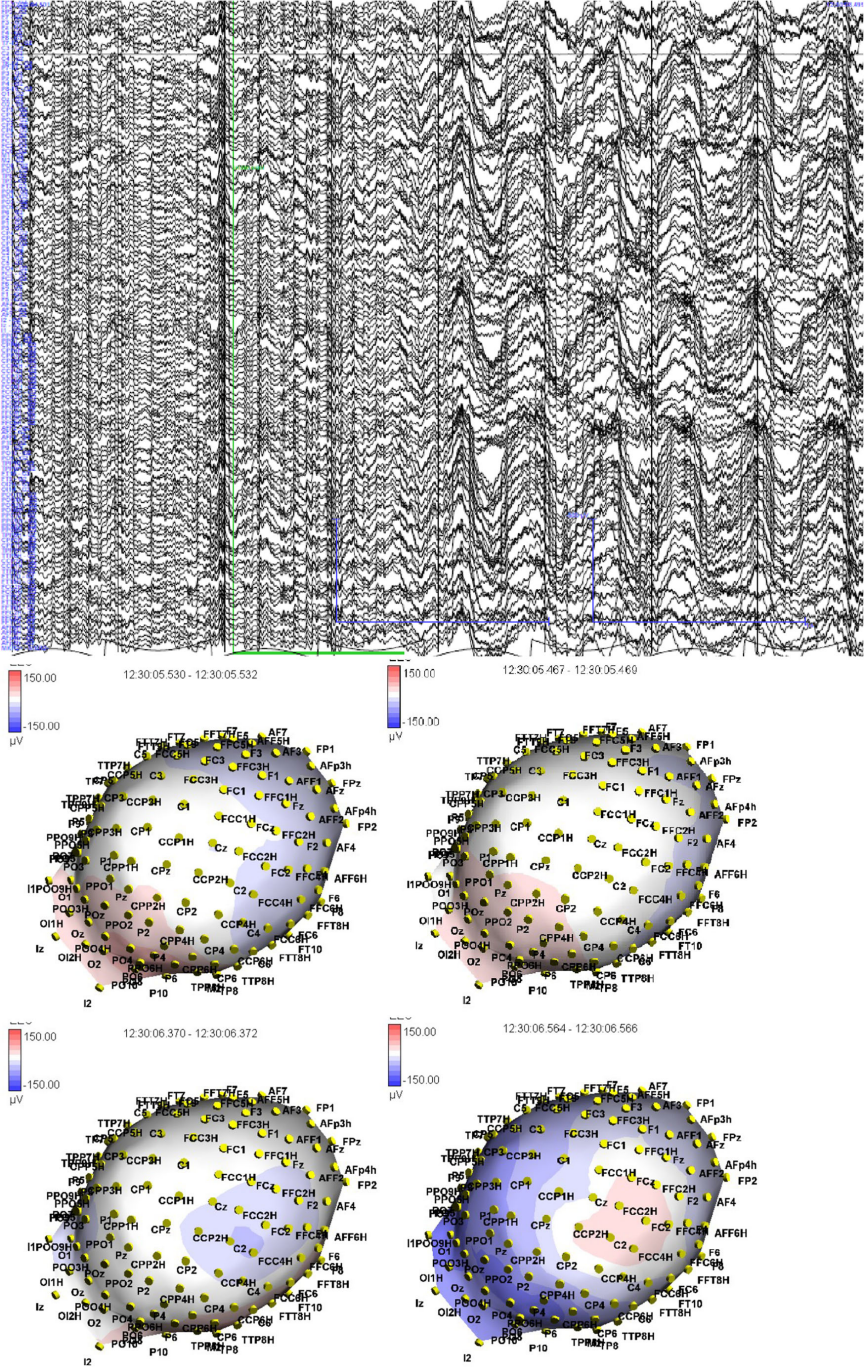
**a** hdEEG epoc selected for the analysis. **b** Map of the amplitudes of interictal discharge on the respective electrodes (duration of the represented interval: 1 s; red refers to maximum positive amplitude, and blue to maximum negative one)

**Fig. 2:**
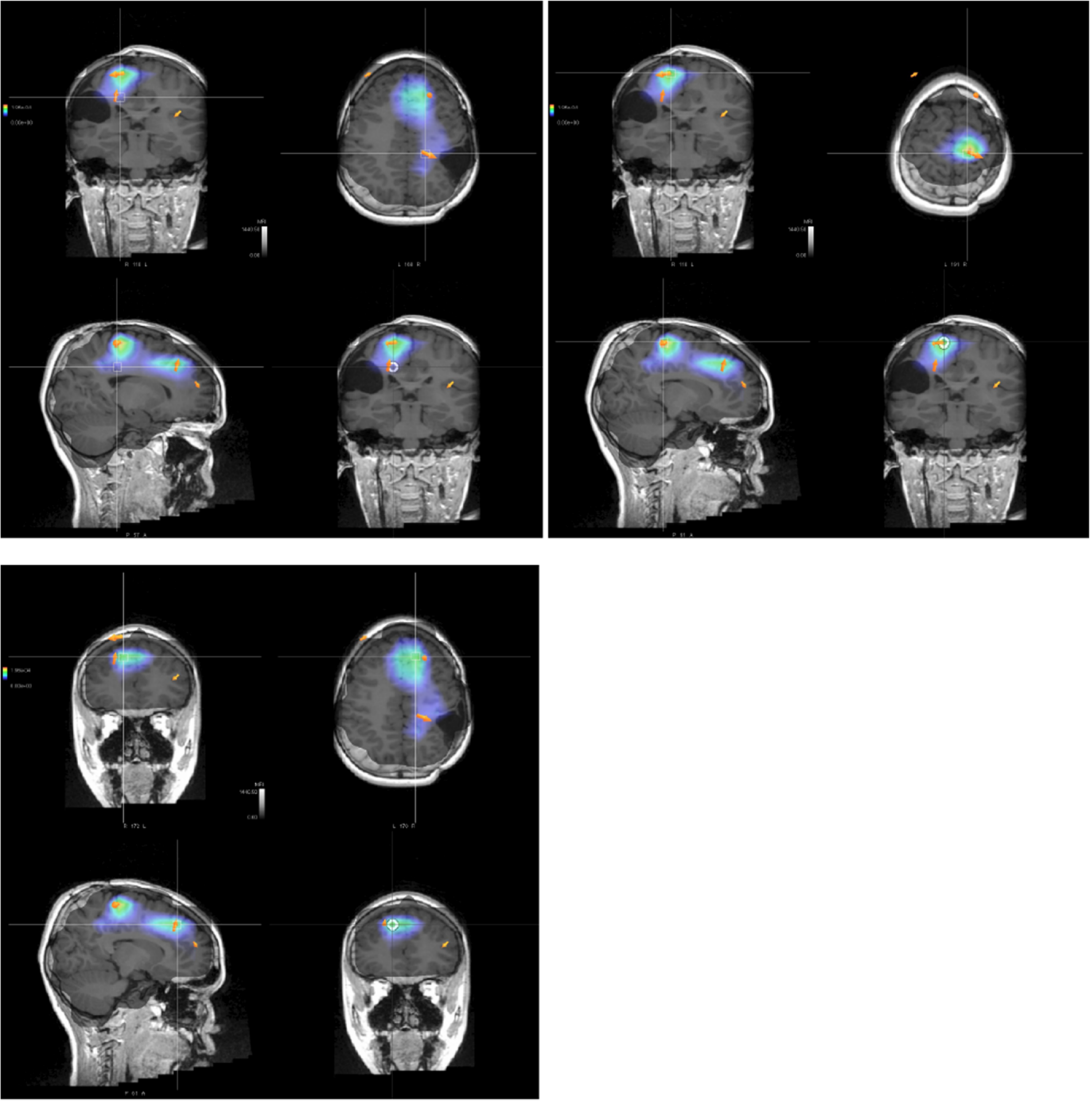
Sources of interictal epileptic discharge of maximal strength (bigger orange arrows), identified through LORETA (blue shadows refer to the areas in which the localization of the source is less probable, vice versa the red ones)

A scalp hdEEG with 128 channels was performed. The registration was made through pre-assembled caps with 128 electrodes according to the 10-10 system (electrode impedances < 40 kΩ; sampling frequency 1 kHz; the vertex was used as recording reference). The recording lasted about 2 h including wake and sleep. No seizures were recorded. Offline, EEG was analyzed through the Micromed SystemView; band pass filtered between 0.1 and 100 Hz and any paroxysmal anomalies was noted. A head model was built using T1-weighted MRI and scalp electrodes were co-registered with the MRI using a template net of electrodes with standard position that was translated/rotated/dilated with personalized digitalization through scalp navigation system (Xensor^TM^ 3D Electrode Digitizer) (Fig. 1). For the inverse solution, low resolution electromagnetic tomography (LORETA) was used through ASA® Experiment Manager® Software. Only the solution point with maximal source strength was taken into account. An interictal source was identified in the mesial part of the right superior parietal lobule. Another source, of lesser force, was found in the right frontal lobe at the level of the right prefrontal area (Fig. 2).

### Stereo electroencephalography (SEEG)

**Fig. 3:**
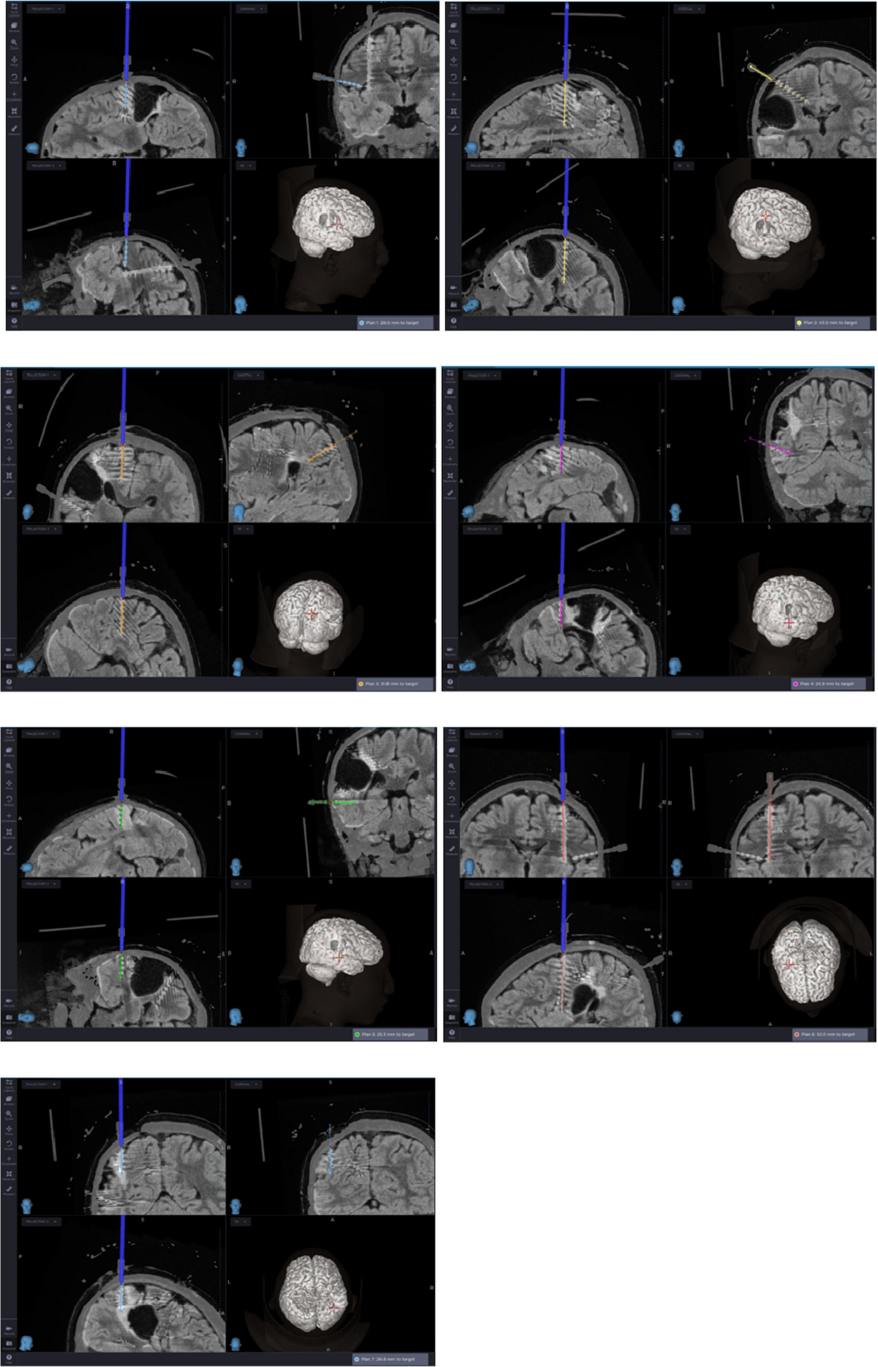
Pre-operative SEEG planning: 1 electrode O; 2 S, 3 Pi, 4 V, 5 T, 6 I, and 7 Ps

SEEG was also performed. Seven depth electrodes were implanted; 4 platinum electrodes; 8 contacts with 5 mm interelectrode spacing, with a total recording surface of 37 mm (Ad-Tech LTM—Spencer probe depth electrodes) and 3 platinum electrodes; and 10 contacts with 5 mm interelectrode spacing, with a total recording surface of 47mm (Ad-Tech LTM—Spencer probe depth electrodes). The Medtronic Stealth Station™ S8 was used to carry out the pre-operative planning using MR and CT angiography with sequences for neuronavigation (Fig. 3). The electrodes were positioned under frameless Medtronic Stealth Autoguide™ cranial robotic guidance platform with the help of anchor bolts.

**Fig. 4:**
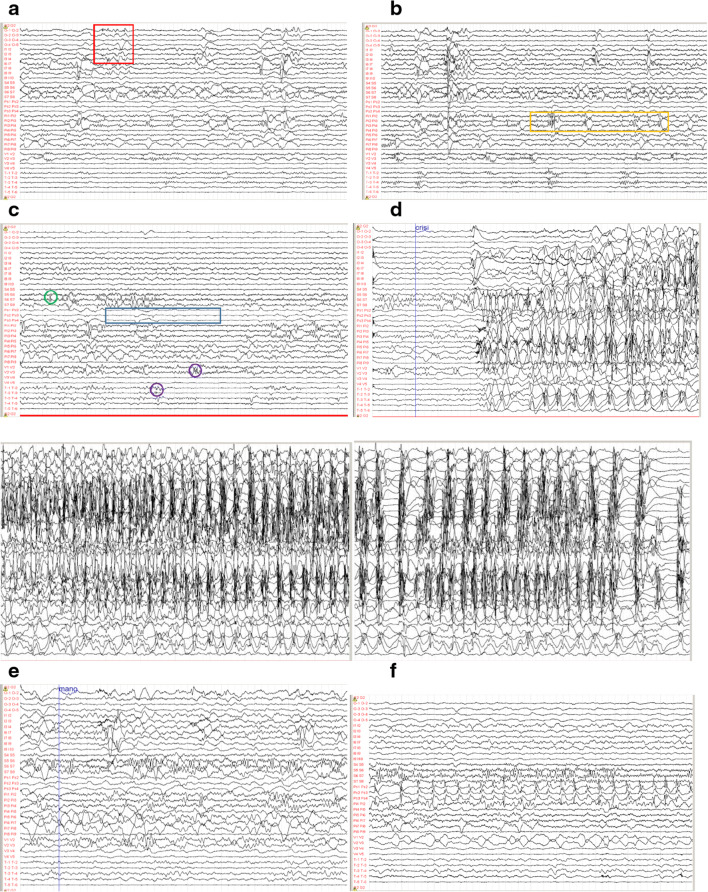
SEEG records. **a**–**c** Interictal paroxysms. **d** Onset and course of focal seizure. **e** Negative myclonus. **f** Electrical seizure on Ps (band pass 0.1–100 Hz; gain 150 μV/cm)

Electrode course description is as follows: O (8 contacts) pars opercularis, S (10) superior parietal lobule, Ps (8) parietal, Pi (10) inferior parietal lobule, V (8) posterior portion of superior parietal gyrus, T (8) median temporal gyrus, and I (10) insula (Fig. 4). From prolonged registration, frequent interictal anomalies were recorded on the deeper contacts of electrode O, I, and on Pi. Less frequent anomalies were found on the more lateral contacts of S and on the deeper ones of T and V. Five focal motor seizures were recorded showing onset on deep O, I, and Pi contacts with slight advance in the first 2 electrodes. Negative myoclonus was related to the presence of fast ripples on the deep contacts of O and I and subsequent slow potential on the most superficial contacts of I. Two electrical seizures were recorded on Ps. Finally, symptoms generally present during patient’s focal non-motor seizures were evoked with the stimulation of the contacts I3 and I4.

## Surgical procedure

After multidisciplinary discussion a surgical approach was proposed to remove the presumed EZ, including pericavitary area, frontal operculum, superior insular, and mesial parietal regions. In general, anesthesia (TIVA/TCI modality with neurophysiological monitoring) reopening of the right temporo-parietal incision and craniotomy was performed. After dural opening, control of the epileptogenic areas identified with SEEG was carried out under magnetic, ultrasound and ECoG navigation. These analyses confirmed the pericavitary area as epileptogenic thus proceeding to its circumferential removal for 1 cm, widening the resection at the level of the posterior parietal area towards the midline. Frontal operculum was identified and removed too under ECoG guidance as well as somatosensory evoked potential and motor evoked potential monitoring. At the end of surgery, a satisfactory ECoG silencing was obtained.

**Fig. 5:**
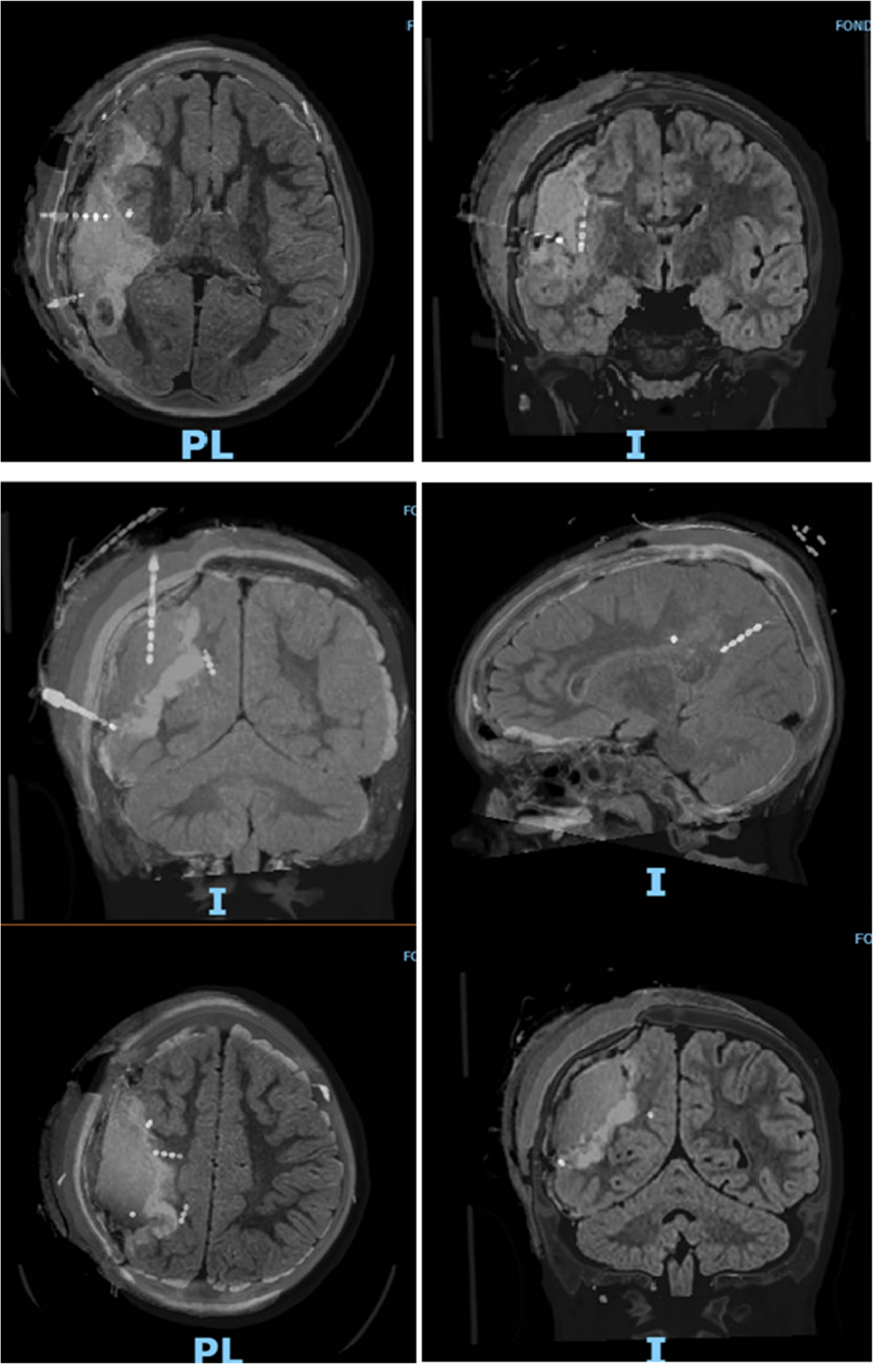
Post-surgical MRI with superimposition of CT sequences for SEEG electrodes localization confirming the removal of the identified epileptogenic areas

Post-operative course was uneventful except for a mild left hemiparesis which gradually recovered during hospitalization. MRI scan showed the extent of resection without complications (Fig. 5).

## Discussion

Surgical resection or complete disconnection of the EZ (cortical area generating clinical seizures) is the objective in patients suffering from drug-resistant focal epilepsy [18]. Several diagnostic tools are available to define indirectly the location and boundaries of EZ, being ESI and radiology the major non-invasive tool [15].

While MRI scan can easily demonstrate epileptogenic lesions, irritative zone (IZ) and seizure onset zone (SOZ) recognition can be more challenging.

Still now, invasive recording remains the gold standard in SOZ definition [22]. In fact, surface EEG can only record brain surface leaving deeper areas potentially unexplored. Moreover, an intrinsic problem of hdEEG rests in the short registration time affordable secondary to the hardness in evaluating such a huge mass of data in longer registrations. However, some studies found that interictal ESI provides an excellent estimate of SOZ [14]. Furthermore, recent studies on high frequency oscillations (HFO, 80–500 Hz) also reinforced hdEEG value in identifying SOZ [10]. HFO are nowadays considered EEG markers for epileptic activity. Furthermore, it appears that HFO-generating brain removal correlates with better post-surgical seizure outcome.

Although these techniques have been increasingly validated, they might fail. Focusing only on the most frequent interictal discharges (IEDs), it is possible to miss SOZ especially whenever it is not the most active IED generator or when IED are multiple. Therefore, recordings and source localization of actual seizures might be very useful especially when interictal ESI is ambiguous or not possible [16]. Directed connectivity analysis is a further promising technique for seizure onset localization, focusing on how strongly each source broadcasts its activity to other brain regions. This approach was proven more accurate than merely considering the most active source [20].

Focusing hdEEG on pediatric patients, a possible issue for clinical use regards superficial electrode number. Papers from the literature state how to reach an adequate level of accuracy and sensitivity for hdEEG in adults, at least 128–256 electrode have to be used [8]. Actually, the largest increment in accuracy occurred when the number of electrodes increased from 31 to 63, with a smaller but measurable gain upon increasing to a 123-electrode system [12]. Particular extra care has to be taken into electrodes montage sampling inferior temporal areas with electrodes around and below the ears and on the cheeks and neck [3, 14]. In children, such a high number of electrodes are not always achievable due to the smallness of the head. In our experience, a maximum of 64–128 channels can be used.

Further problems regarding ESI in children are related to multifocal and diffuse IEDs patterns even in cases of focal epilepsy with a delimited EZ. Analyzing the literature, it seems that in children non-epileptiform abnormalities may be accurate in localizing abnormal cortical function than spiking activity [17]. Similarly, intermittent or continuous focal slow waves on scalp EEG can represent a sign of focal dysfunction potentially associated with an epileptogenic lesion or even represent an ictal pattern. Therefore, ESI of focal slowing could be a useful to localize EZ in children complementary to the ESI of IEDs [1].

In order to evaluate these problematic, the authors presented the case previously described hoping to underline potential roles for hdEEG in future prospective. In fact, the heterogeneous seizures phenomenology of the child and the lack of video EEG localization failed to give sure targets for surgery. hdEEG and interictal recording analysis, together with clinical data, suggested that the pericavitary area as well as right frontal lobe were involved in seizures onset. These findings also helped to direct the positioning of the SEEG electrodes confirming the presence of epileptic discharges coming from the frontal operculum as well as from the more intuitive parietal EZ. Coming finally to surgical resection, merging hdEEG, SEEG, and ECoG was possible to obtain electrical silence after removal of the defined EZ. The role of hdEEG in our patient’s pre-surgical workup was in agreement with Foged et al. that observed ESI through hdEEG had additional value in one third of the patients analyzed [9]. Mégevand et al. reached similar conclusions considering the localization of interictal spikes through hdEEG as a good estimate of seizure onset zone and therefore recommending to consider ESI in the planning of intracranial recording [15].

One of the most important debates about ESI is whether hdEEG could identify deep sources. In fact, in a large study that included 152 patients [5], few cases failed in ESI, showing that propagated interictal epileptiform discharges were used while deep recordings revealed the real sources. It is presumable that this also happened in our case, as SEEG highlighted the frontal operculum and the anterior part of the insula as the sources of the seizures. However, having merged EZ source in the right prefrontal area via hdEEG with clinical data, a greater coverage of the intracranial recording was planned. It is possible that by analyzing the HFOs in the hdEEG recording, a better localization of the SOZ could have been achieved; however, this was not possible in our case due to the presence of technical issues. This combination of non-invasive tools could be even more important in pediatric epilepsy surgery due to the difficulties in obtaining SEEG in very young and non-collaborative patients.

Finally, analyzing the aforementioned data it was possible to see how hdEEG and ECoG offered a superficial and depth covering of the epileptic discharges overlapping SEEG one. This approach that integrates the information obtained during the pre-surgical workup with the ECoG seems unprecedented as no studies are published in the medical literature.

## Conclusion

ESI represent a relevant tool in epilepsy surgery workup in order to define EZ together with clinical data, MRI, neuropsychological assessment, and video EEG. ESI via hdEEG increases the localizing power of surface electroencephalographic recordings especially when coupled with intraoperative ECoG, granting a more easily identification of deep sources. This combination might even in the future grant the possibility to further reduce the need for SEEG in selected cases, which is of utmost importance in the pediatric setting and in particular in very young patients.
